# Preterm Birth Affects Early Motor Development in Pigs

**DOI:** 10.3389/fped.2021.731877

**Published:** 2021-10-07

**Authors:** Charlotte Vanden Hole, Miriam Ayuso, Peter Aerts, Steven Van Cruchten, Thomas Thymann, Per Torp Sangild, Chris Van Ginneken

**Affiliations:** ^1^Laboratory of Comparative Perinatal Development, Department of Veterinary Sciences, Faculty of Biomedical, Pharmaceutical and Veterinary Sciences, University of Antwerp, Antwerp, Belgium; ^2^Laboratory of Functional Morphology, Department of Biology, Faculty of Sciences, University of Antwerp, Antwerp, Belgium; ^3^Comparative Pediatrics and Nutrition, University of Copenhagen, Copenhagen, Denmark

**Keywords:** locomotion, prematurity, pig, birth weight, spatio-temporal gait analysis

## Abstract

**Background:** Preterm infants frequently show neuromotor dysfunctions, but it is not clear how reduced gestational age at birth may induce developmental coordination disorders. Advancing postnatal age, not only post-conceptional age, may determine neuromuscular development, and early interventions in preterm newborns may improve their later motor skills. An animal model of preterm birth that allows early postnatal detection of movement patterns may help to investigate this hypothesis.

**Methods:** Using pigs as a model for moderately preterm infants, preterm (106-day gestation, equivalent to 90% of normal gestation time; *n* = 38) and term (115-day gestation, equivalent to 99% of normal gestation time; *n* = 20) individuals were delivered by cesarean section and artificially reared until postnatal day 19 (preweaning period). The neuromotor skills of piglets were documented using spatiotemporal gait analyses on video recordings of locomotion at self-selected speed at postnatal age 3, 4, 5, 8, and 18 days. Results were controlled for effects of body weight and sex.

**Results:** Both preterm and term piglets reached mature neuromotor skills and performance between postnatal days 3–5. However, preterm pigs took shorter steps at a higher frequency, than term piglets, irrespective of their body size. Within preterm pigs, males and low birth weight individuals took the shortest steps, and with the highest frequency.

**Conclusion:** Postnatal development of motor skills and gait characteristics in pigs delivered in late gestation may show similarity to the compromised development of gait pattern in preterm infants. Relative to term pigs, the postnatal delay in gait development in preterm pigs was only few days, that is, much shorter than the 10-day reduction in gestation length. This indicates rapid postnatal adaptation of gait pattern after reduced gestational age at birth. Early-life physical training and medical interventions may support both short- and long-term gait development after preterm birth in both pigs and infants.

## Introduction

Preterm birth (birth before 37 weeks of gestation) is known to interrupt brain growth and maturation *in utero*, potentially affecting postnatal neurodevelopment, especially of cerebellar and periventricular brain structures (1, 2). In addition, clinical complications associated with preterm birth (such as hypoxia, ischemia, inflammation) can further hinder postnatal neurodevelopment (2). Thus, it is not surprising to note high prevalence of neuromotor dysfunctions and poor movement coordination in preterm infants (1, 3–5). Even for infants not diagnosed with specific brain defects (e.g., cerebral palsy) and having normal intelligence, preterm birth may result in motoric challenges, as evidenced by 20–40% incidence of moderate motor impairments, developmental coordination disorders, and neurological dysfunctions (1, 3, 4, 6). Preterm birth is a multifactorial syndrome that requires support at many levels, including the need to facilitate optimal neurodevelopmental outcomes. However, in order to support evidence-based interventions, more basic data on locomotion in preterm newborns are required. In many aspects, preterm pigs born at 90% gestation have proven to be an excellent brain model for preterm infants (7), in addition to aspects of gut and nutritional functions (8). The preterm pig as a model for neuromuscular development in preterm infants is less explored. In contrast to the immediate locomotion after birth of term piglets, 90% gestation preterm pigs show 3–5-day delays in their normal standing and walking (9–11). While this postnatal motor development is much faster than in preterm infants, the preterm–term differences in piglets may be used to investigate basic mechanisms and possible interventions in states of immaturity. Further, the gross anatomy of the brain (12), its perinatal growth and developmental trajectories are similar in pigs and infants (13), suggesting that consequences of preterm birth may be similar, although different in their timing (8, 14). Specifically, for locomotion, it appears that underlying mechanisms of development and locomotor modules in the neuronal networks of the spinal cord are similar among mammals (15–18).

Previous studies on motor skills in preterm pigs showed that more days were required to achieve basic motor skills (time to first stand and walk), with lower overall physical activity level, compared with term pigs (9–11). Similarly, in preterm infants delayed or absent physical movement predict a delay in onset of first walking (6) and walking coordination (1, 19). A previous study on gait development at 1 week of age in preterm and term pigs revealed only minor differences, although shorter strides and step lengths were present in preterm pigs after normalization for their smaller size (9). Interestingly, shorter strides are also observed in preterm infants, although such effects may disappear at later ages (1, 6). No longitudinal postnatal locomotion studies comparing preterm and term counterparts are available in pigs or infants. Studies in preterm pigs show that organ systems respond widely different to reduced gestational age at birth, either with fast adaptation after birth (lungs, gut, immunity) or with more prolonged defects, related to their immature state at birth (e.g., brain, bone, metabolism) (8, 20). Whether postnatal, rather than post-conceptional age, is the main driver of neuromuscular maturation is unclear. Rapid postnatal adaptation of neuromuscular development would encourage specialized neonatal support to reduce later dysfunctions (6, 21, 22).

The present study compares motor skill development in piglets that are born preterm (90% gestational age) (P) and term (T) at different time-points: 3, 4, 5, 8, and 18 days postnatally. We compare different aspects of gait between both groups and along the set of time-points: motor performance (speed, stride length, stride frequency), neuromotor skill/maturation (normalized motor performance and the different components of a stride cycle, for example, normalized step length, duty factor), and gait variability [for more info, see **Materials and Methods** section and previous publications (23, 24). We hypothesize that postnatal age rather than post-conceptual age drives gait development. Specifically, we hypothesize that preterm pigs show no developmental delay in motor performance, an equally long period of neuromotor maturation and a similar gait variability compared to pig born near term. Considering that low birth weight and sex have been reported to affect morbidities and motor development in infants (4, 5, 25–27) and preterm pigs (10, 20), we made subgroup analyses according to weight (0–25 vs. 50–75% birth weight percentiles) and sex (male, female).

## Materials and Methods

### Study Animals

Four litters of pigs (Danish Landrace × Large White × Duroc) (litter sizes 20–23) were used in this study. One litter was born by cesarean section at term (gestation day 115; term = 115–117 days, T piglets, *n* = 20, birth weight (live born piglets) 1,049 ± 222 g). Three other litters were delivered preterm at 90% gestation through a cesarean section (gestation day 106; P piglets, *n* = 38, birth weight (live born piglets) 883 ± 199 g), as outlined previously (14). In brief, the piglets were resuscitated immediately after cesarean section and placed in oxygenated and temperature-controlled incubators. Within 3 h after birth, a catheter was placed *via* the transected umbilical artery to allow parenteral nutrition support, and an orogastric feeding tube was placed to allow enteral bolus feeding. The postnatal handling of these two groups was identical and followed the protocol outlined in (14). Both T and P piglets were nourished enterally with increasing amounts of raw bovine milk with lactose added (Variolac, 6 g/l, Arla Foods Ingredients, Århus, Denmark). During the first 7 days of life, the pigs were fed with parenteral nutrition (modified Kabiven solution, Fresenius Kabi, Bad Homburg, Germany) in addition to their enteral diet. All the piglets received the same amount of nutrition (relative to body weight). The clinical condition of each piglet was evaluated at least twice per day. During the first 5 days of life, these piglets were kept individually in heated incubators with supplemental oxygen during the first 12 h after birth. Later, they were transferred to larger open cages. P and T piglets were not mixed.

All piglets were euthanized on postnatal day 19 using initial induction of anesthesia (mixture of zolazepam, tiletamine, ketamine, butorphanol, and xylacin) followed by intracardiac injection of a lethal dose of sodium pentobarbital. All experimental procedures were approved by the Danish Animal Experiments Inspectorate (2014-15-0201-00418).

### Video Sequences

Piglets were gently encouraged to walk at voluntary speed through a custom-made corridor (fitted with a reference grid), while lateral view video recordings were made (12.8-megapixel, 50 Hz, JVC GC-PX100, JVC Kenwood Corporation, Kanagawa, Japan) at five time points: 3, 4, 5, 8, and 18 days after birth. At each recording day, three successive movies per piglet were recorded. Only videos that had at least one completed cycle without pausing or falling were retained for further analysis. In each retained sequence, one complete stride cycle was analyzed. More details on the recording and selection of the video sequences are found in previous papers (23, 24).

### Gait Analysis

In each of the video sequences, five body landmarks were digitized field-by-field using Matlab (MathWorks, Natick, MA, USA), using a free work package written by Ty Hedrick (University of North Carolina, USA; http://www.unc.edu/~thedrick/software1.html). The first four points were the most distal point of the distal phalanx (claw of the fourth toe) of each leg, and the fifth point was the eye or a dark spot on the skin. The latter body point was included to measure overall forward displacement of the body throughout a stride. An image showing the setup of the recording has been previously published by our group and depicts all the reference points used in the gait analysis (23). From our previous gait studies, we know that the choice of the landmark does not introduce extra variability to the dataset, as long as the landmarks are easily tracked throughout the stride (23, 24).

A linear dimension—related to locomotion—was necessary for the normalization of certain variables [dynamic similarity principle by Alexander and Jayes (28); see Table 1]. In previous gait analysis of the piglet, functional hind limb length (HLL), that is, the distance between the most distal part of the distal phalanx (of the fourth toe of the hindlimb) and the tail base, was used (23, 24). HLL was measured once in each sequence, in the frame where the hind limb closest to the camera was on the floor and supporting the piglet's weight. In addition, two points on the reference grid were digitized for scaling purposes (for more information on the setup and an image depicting all these landmarks, we refer the reader to (23).

**Table 1:** Summary of all used variables (abbreviations (ABB), definitions, and formulas, including normalization procedure [NP, adapted from (23)].

**Variable**	**ABB**	**Definition**	**Formula**	**NP**
Gravitational acceleration	*g*	–	–	–
Self-selected speed	*u*	The forward movement during one cycle divided by the duration of the cycle. Animals are able to move in an unrestrained, voluntary way.	*fl*_*stride*_	$\frac{u}{\sqrt{HLL\;g}}$
Stride frequency	*f*	Inverse of the period between two consecutive footfalls of a certain leg.	$\frac{u}{l_{stride}}$	$f\;\sqrt{HLL\;g}$
Stride length	*l*_stride_	The forward movement during one stride or cycle.	$\frac{u}{f}$	$\frac{l_{stride}}{HLL}$
Stance duration	*t_*st*_*	The period of contact between a limb and the ground.	–	$\frac{t_{st}}{\sqrt{HLL/g}}$
Swing duration	*t*_sw_	The period of limb flight.	–	$\frac{t_{sw}}{\sqrt{HLL/g}}$
Step length	*l*_step_	The forward movement during one step (stance phase only).	–	$\frac{l_{step}}{HLL}$
Duty factor	*df*	The fraction of the cycle for which the limb is in contact with the ground.	–	NA
Maximum swing height	*h*_swmax_	The maximum vertical distance the leg is lifted from the ground during the swing phase.	–	$\frac{h_{swmax}}{HLL}$
AI stride frequency	AIF	Asymmetry index of the stride frequency. Adapted from (29).	$\frac{\left(f_{R}\;-\;f_{L}\right)}{0.5\;\left(f_{R}+f_{L}\right)}\;100\%$	–
AI stride length	AIL	Asymmetry index of the stride length. Adapted from (29).	$\frac{\left(l_{stride,\;R}\;-\;l_{stride,L}\right)}{0.5\;\left(l_{stride,R}+\;l_{stride,L}\right)}\;100\%$	–
AI stance duration	AIST	Asymmetry index of the stance duration. Adapted from (29).	$\frac{\left(t_{st,R}\;-\;t_{st,L}\right)}{0.5\;\left(t_{st,R}+t_{st,L}\right)}\;100\%$	–
AI swing duration	AISW	Asymmetry index of the swing duration. Adapted from (29).	$\frac{\left(t_{sw,R}\;-\;t_{sw,L}\right)}{0.5\;\left(t_{sw,R}+t_{sw,L}\right)}\;100\%$	–
AI step length	AISL	Asymmetry index of the step length. Adapted from (29).	$\frac{\left(l_{step,R}\;-\;l_{step,L}\right)}{0.5\;\left(l_{step,R}+l_{step,L}\right)}\;100\%$	–
AI duty factor	AIDF	Asymmetry index of the duty factor. Adapted from (29).	$\frac{\left(df_{R}\;-\;df_{L}\right)}{0.5\;\left(df_{R}+df_{L}\right)}\;100\%$	–

*When normalized, variables are indicated with ′ in the text*.

Fourteen gait variables were calculated using a custom-written Matlab script [made by Goyens, see (23)], based on the digitization of the abovementioned five body landmarks. An overview of all variables, including definitions, formulas, and normalizations (if applicable), is found in Table 1. Variables can be subdivided into three main categories: motor performance, neuromotor skill/maturation, and gait variability (23, 24). Motor performance included absolute values of self-selected speed (*u*) and its components stride frequency (*f*) and stride length (*l*_stride_). Neuromotor skill/maturation included all spatiotemporal gait variables that were normalized to HLL (made dimensionless), in accordance with the dynamical similarity put forth by Alexander and Jayes (28). The evolution of these variables over time indicates neuromotor maturation, whereas differences in these variables between groups indicate a difference in neuromotor skill as such. Gait variability was measured through asymmetry indices (AIs). The smaller the AI, the larger the symmetry. Theoretically, they can range from 200 to 0% (30). Variables were calculated per piglet (*u* and *u*′), per leg pair (AIs) or per leg (spatiotemporal gait variables, both absolute and normalized).

### Statistics

Generalized regression models were used to test whether condition [T (*n* = 20) or P (*n* = 38)] and age after birth (and their interaction) had a significant effect on the different outcome variables. When constructing the models, it was considered whether a variable was calculated per pig (body weight, HLL, u, u′), leg (all absolute and normalized spatiotemporal gait variables with the exception of u and u′), or legpair (AIs). In case of variables that were leg or legpair specific, leg or legpair was added as a fixed effect, and a random factor for leg or legpair nested in piglet could be added (when proven a significant addition to the model through log-likelihood testing). *Post hoc* testing was approached differently, depending on the investigated effect and variable. When comparing legs, *post hoc* testing with Tukey's correction was applied. In case of an age effect, *post hoc* testing with Dunnett's correction was applied. In this case, day 18 (as the most mature age) was used as a reference, as this reduced the number of between-group comparisons and because we were interested in the maturation pattern and not the day-to-day-variation.

The testing of P-MALE (*n* = 21) vs. P-FEMALE (*n* = 17) pigs was done on the entire preterm dataset. For selecting which piglets were P-LBW and P-NORM, per litter the lowest-quartile (0–25th percentile) and the third-quartile (50–75th percentile) birth weights were calculated. This led to a P-LBW group of nine filmed piglets (birth body weight 660 ± 251 g) and a P-NORM group of nine filmed piglets (birth body weight 990 ± 130 g). The statistical analysis itself was the same as in the T vs. P dataset, with “condition” being replaced by “BW-category” or “sex.” Interactions between these two effects could not be included in the model, because the P-LBW/P-NORM dataset was too small to include sex as an effect.

## Results

### Morphometrics

There was no interaction between condition (P vs. T), sex or birth weight and postnatal age for both body weight, and HLL (*p* > 0.05). Thus, only the overall effects of age and gestational age were statistically assessed.

The body weight of the piglets in the age groups 3, 4, 5, and 8 days was significantly different from that at the reference age d18 [*p* < 0.001 for P (*n* = 38), T (*n* = 20), P-LBW (*n* = 9), P-NORM (*n* = 9), P-MALE (*n* = 21), and P-FEMALE (*n* = 17)] (Table 2). Additional *post-hoc* analysis—comparing all age groups with each other—revealed that the body weight of P and T piglets (as well as P-LBW, P-NORM, P-MALE, and P-FEMALE) remained constant from d3 up to and including d8 and increased between d8 and d18 (*p* < 0.001 for d3–d8 vs. d18). Overall, body weight was lower in P compared to T piglets (*p* = 0.001). Body weight was overall lower in P-LBW piglets compared to P-NORM piglets (*p* < 0.001) but did not differ between P-MALE and P-FEMALE.

**Table 2:** Body weight (mean ± SD; kg) according to gestational age at birth (condition) (preterm at gestational age 106 days—term at gestation age 115 days) and postnatal age (3, 4, 5, 8, and 18 days), birth weight (low birth weight (LBW): 0–25th percentile—normal birth weight (NORM): 50–75th percentile), and sex (female vs. male).

	**Effect**					
	**Condition**		**Birth weight (preterms)**		**Sex (preterms)**	
**Age**	**Preterm**	**Near term**	**LBW**	**NORM**	**Female**	**Male**
d3	0.90 ± 0.22^a^	1.03 ± 0.23^a^	0.65 ± 0.18^a^	0.93 ± 0.77^a^	0.99 ± 0.20^a^	0.84 ± 0.22^a^
d4	0.87 ± 0.14^a^	1.08 ± 0.24^a^	0.68 ± 0.19^a^	0.91 ± 0.77^a^	0.89 ± 0.19^a^	0.88 ± 0.12^a^
d5	0.98 ± 0.23^a^	1.08 ± 0.24^a^	0.73 ± 0.21^a^	0.96 ± 0.11^a^	0.99 ± 0.20^a^	0.95 ± 0.25^a^
d8	1.05 ± 0.26^a^	1.13 ± 0.24^a^	0.77 ± 0.21^a^	1.11 ± 0.22^a^	1.11 ± 0.27^a^	1.02 ± 0.26^a^
d18	1.73 ± 0.28^b^	1.57 ± 0.30^b^	1.54 ± 0.11^b^	1.82 ± 0.24^b^	1.69 ± 0.30^b^	1.77 ± 0.27^b^

*There was no interaction between condition, sex, or birth weight and postnatal age*.

a,b *Different superscripts indicate significant differences with postnatal age within condition, birth weight, or sex*.

HLL in each age group was significantly lower from that at the reference age d18 [*p* < 0.0001 for P (*n* = 38), T (*n* = 20), P-LBW (*n* = 9), P-NORM (*n* = 9), P-MALE (*n* = 21), and P-FEMALE (*n* = 17)] (Table 3). Additional *post-hoc* analysis—comparing all age groups with each other—revealed HLL remained constant from d3 up and including d5 and increased between d5 over d8 to d18. In addition, P piglets had shorter legs, as evidenced by a shorter HLL (*p* < 0.001). HLL was overall lower in P-LBW piglets compared to P-NORM piglets (*p* < 0.001) but did not differ between P-MALE and P-FEMALE.

**Table 3:** Hindlimb length (HLL) (mean ± SD; m) according to gestational age when cesarean-section derived (condition) (preterm at gestational age 106 days—term at gestation age 115 days) and postnatal age (3, 4, 5, 8, and 18 days), birth weight (low birth weight (LBW): 0–25^th^ percentile—normal birth weight (NORM): 50–75th percentile), and sex (female vs. male).

	**Effect**					
	**Condition**		**Birth weight (preterms)**		**Sex (preterms)**	
**Age**	**Preterm**	**Near term**	**LBW**	**NORM**	**Female**	**Male**
d3	0.14 ± 0.02^a^	0.15 ± 0.01^a^	0.13 ± 0.02^a^	0.14 ± 0.01^a^	0.15 ± 0.01^a^	0.14 ± 0.02^a^
d4	0.14 ± 0.01^a^	0.16 ± 0.01^a^	0.13 ± 0.01^a^	0.14 ± 0.01^a^	0.14 ± 0.01^a^	0.14 ± 0.01^a^
d5	0.14 ± 0.01^a^	0.15 ± 0.01^a^	0.13 ± 0.01^a^	0.15 ± 0.01^a^	0.15 ± 0.01^a^	0.14 ± 0.02^a^
d8	0.16 ± 0.02^b^	0.17 ± 0.01^b^	0.14 ± 0.02^b^	0.16 ± 0.01^b^	0.16 ± 0.02^b^	0.15 ± 0.02^b^
d18	0.18 ± 0.02^c^	0.17 ± 0.01^c^	0.16 ± 0.01^c^	0.18 ± 0.01^c^	0.17 ± 0.02^c^	0.18 ± 0.01^c^

*There was no interaction between condition, sex, or birth weight and postnatal age. HLL was lower in P as compared to T piglets and in P-LBW piglets compared to P-NORM piglets*.

a−c *Different superscripts indicate significant differences with postnatal age within condition, birth weight, or sex*.

### Motor Performance

**Figure 1:**
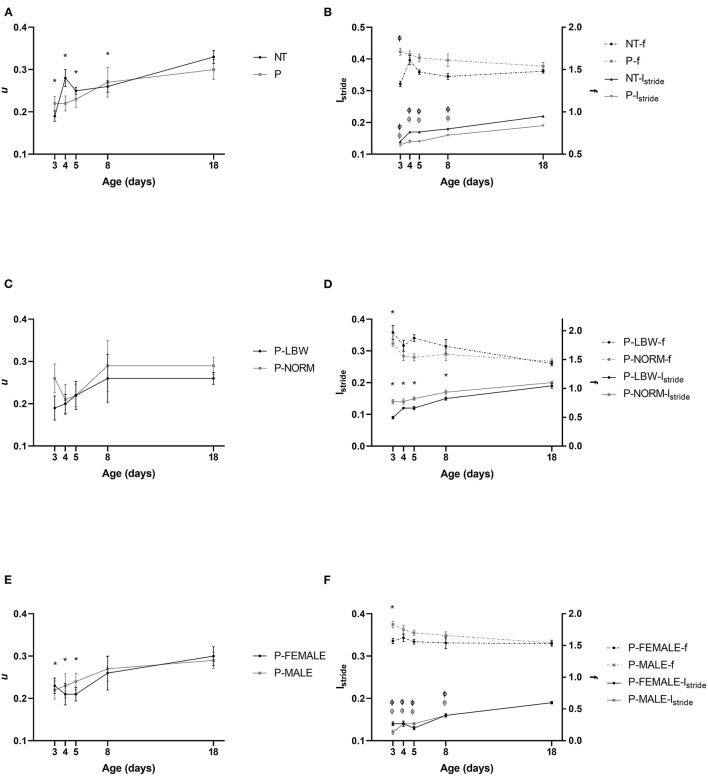
Motor performance. All values are mean ± SE. **(A)** Speed (*u*, ms^−1^) according to condition (P: preterm, gray; T: term, black) and age. *u* of P and T piglets is similar. Mean values indicated with * differ from d18. **(B)** For stride length (*l*_stride_, m, full line) and stride frequency (*f*, s^−1^, dotted line), an interaction was observed between condition (P vs. T) and postnatal age. *l*_stride_ was higher in T than in P pigs. *f* was lower in T, compared to P pigs at d3, d5, and d8). Mean values of *l*_stride_ indicated with “Φ” differ from d18 for P and T. Except for T piglets increasing their *f* from d3 to d18, T and P pigs did not change their *f* with age. **(C)** Speed (*u*, ms^−1^) of preterm pigs did not differ with birth weight (normal birth weight (P-NORM); low birth weight (P-LBW)) and postnatal age. **(D)** Stride length (*l*_stride_, m, full line) and stride frequency (*f*, s^−1^, dotted line) according to birth weight (P-NORM vs. P-LBW) and postnatal age. P-NORM piglets had a higher *l*_*stride*_ and a lower *f*, compared to P-LBW. Mean values indicated with * differ from d18 for *l*_stride_ and for *f*. **(E)** Speed (*u*, ms^−1^) according to sex of preterm piglets (female: P-FEMALE; male: P-MALE) and age. No differences are noted with sex. Mean values indicated with * differ from d18. **(F)** Stride length (*l*_stride_, m, full line) and stride frequency (*f*, s^−1^, dotted line) according to sex (P-MALE vs. P-FEMALE) and postnatal age. There was an interaction of postnatal age and sex for *l*_*stride*_, indicating that *l*_stride_ was higher for P-FEMALE at d3 whereas the effect for *f* was not depending on postnatal age and *f* was overall higher in P-MALE. Mean values of indicated with “Φ” and “*” differ from d18 for *l*_stride_ and *f*, respectively. Black colors indicate differences in T while gray colors indicate differences in P piglets.

*u* did not show any interaction between condition (P vs. T) and postnatal age and was not different between P (*n* = 38) and T (*n* = 20) piglets. *u* increased with postnatal age (*p* < 0.001). Irrespective of P or T, piglets at d3–8 had a significantly lower *u* compared to d18 (d3: *p* < 0.001, d4: *p* = 0.010, d5: *p* = 0.002, d8: *p* = 0.029) (Figure 1A) (data points = 215). As for the components of *u*, we did note an interaction between condition and age (*l*_*stride*_: *p* < 0.001; *f* : *p* = 0.002) resulting in differences between P and T piglets. *l*_*stride*_ in both groups increased up to d18 (*p* < 0.001 for all group comparisons). At every time point, *l*_*stride*_ was higher for T piglets, compared to P piglets [*p* = 0.010 (d3), *p* < 0.001 (d4–18)]. T piglets increased their *f* from d3 to d18 (*p* = 0.021), but other age differences were not observed in both T and P piglets. *f* was significantly lower in T, compared to P piglets at d3, d5, and d8 (*p* < 0.001, *p* = 0.001, *p* = 0.024, respectively) (Figure 1B) (data points = 860). This means that T piglets took bigger steps at a lower frequency than P piglets, resulting in a similar speed.

*u* was not different for P-LBW (*n* = 9) and P-NORM (*n* = 9) piglets (Figure 1C) (data points = 60). Similar to T vs. P piglets, this *u* was achieved differently: P-NORM piglets had a higher *l*_*stride*_ and a lower *f*, compared to P-LBW (*p* < 0.001 and *p* = 0.016, respectively). *l*_*stride*_ was increased (all comparisons with d18 *p* < 0.001), whereas *f* was dropped with age (only d3 differed from d18, *p* < 0.001) (Figure 1D) (data points = 240).

P-MALE (*n* = 21) and P-FEMALE (*n* = 17) piglets did not differ in *u* (Figure 1E) (data points = 134), although *l*_*stride*_ was higher for P-FEMALE at d3 (*p* < 0.001) and *f* was overall higher in P-MALE (*p* = 0.001). Both groups increased their *l*_*stride*_ (all comparisons with d18 *p* < 0.001) and overall dropped their *f* (only d3 differed from d18, *p* < 0.001) (Figure 1F) (data points = 536).

### Neuromotor Control—Normalized Motor Performance

**Figure 2:**
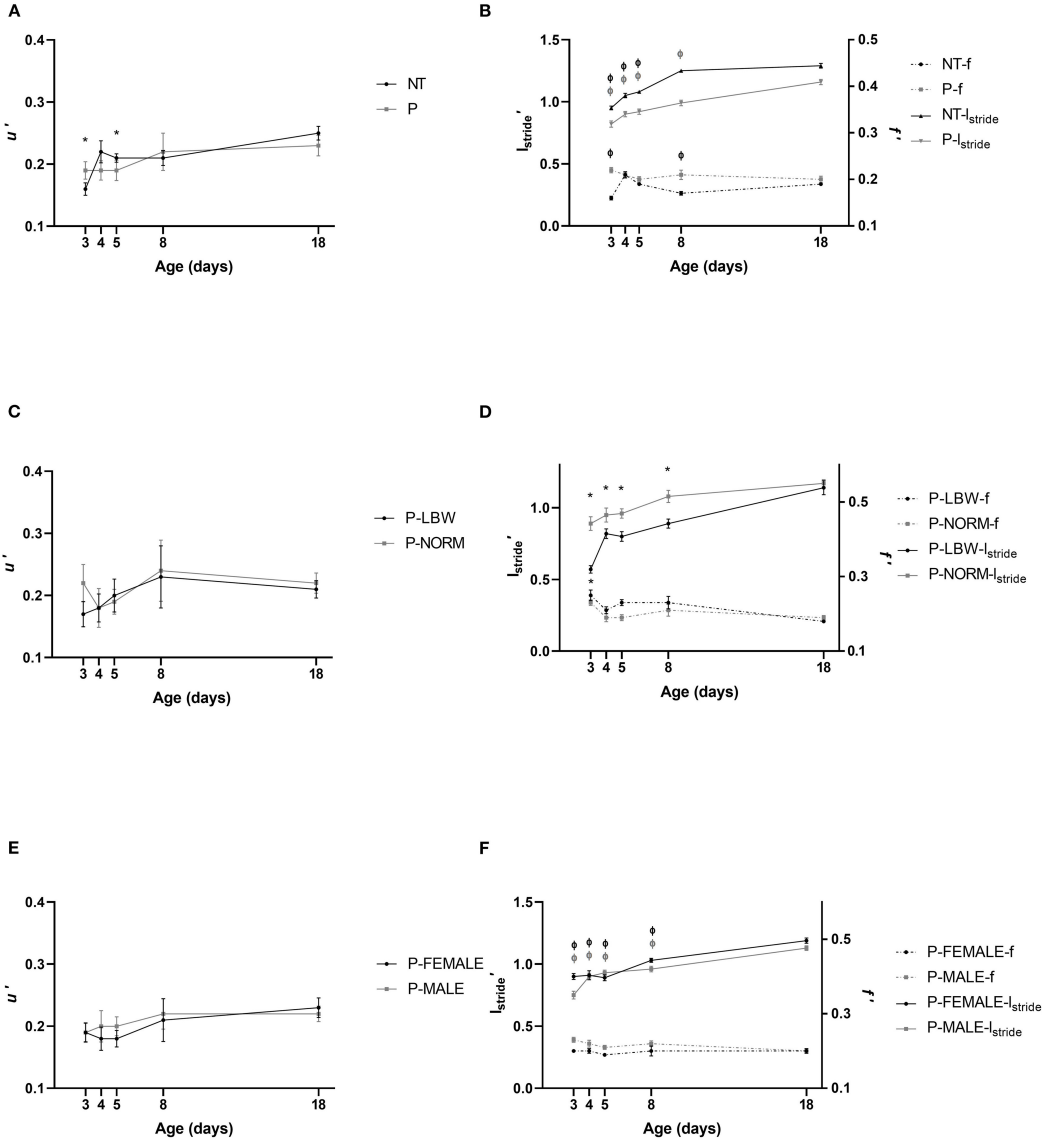
Neuromotor control. All values are mean ± SE. **(A)** Normalized speed (*u*′) according to condition (P: preterm, gray; T: term, black) and age. *u*′ of P and T piglets is similar. Mean values indicated with * differ from d18. **(B)** For normalized stride length (*l*′_stride_, full line) and stride frequency (*f*′, dotted line), an interaction was observed between condition (P vs. T) and postnatal age. *l*_stride_′ was shorter in P piglets. At d3, d5, and d8, *f*′ was higher in P piglets, compared to T piglets. Mean values of *l*_stride_′ and *f*′ indicated with “Φ” were different from d18. **(C)** Normalized speed (*u*′) of preterm pigs did not differ with birth weight (normal birth weight (P-NORM); low birth weight (P-LBW) and postnatal age. **(D)** Normalized stride length (*l*′_stride_, full line) and normalized stride frequency (*f*′, dotted line) according to birth weight (P-NORM vs. P-LBW) and postnatal age. *l*_stride_′ was higher for P-NORM, and *f*′ was overall higher for P-LBW. Mean values of *l*_stride_′ and *f*′ indicated with “*” were different from d18. **(E)** Normalized speed (*u*′) did not differ according to sex of preterm piglets (female: P-FEMALE; male: P-MALE) and postnatal age. **(F)** Normalized stride length (*l*′_stride_, full line) and normalized stride frequency (*f*′, dotted line) according to sex (P-MALE vs. P-FEMALE) and postnatal age. There was an interaction of postnatal age and sex for *l*′_*stride*_ indicating that *l*_stride_ was higher for P-FEMALE at d3, 8, and 18, while *f*′ was overall higher in P-MALE. Mean values of indicated with “Φ” differ significantly from d18 for *l*′_stride_. Black colors indicate differences in T while gray colors indicate differences in P piglets.

*u*′ was not different between P (*n* = 38) and T (*n* = 20) piglets and increased in a similar fashion. Irrespective of P or T, *u*′ was lower at d3 and d5, compared to d18 (*p* = 0.001, 0.050, respectively), indicating that the piglets were able to obtain a mature normalized speed between d5 and d8 (Figure 2A) (data points = 215). For *l*_stride_′, the maturation period was longer in P piglets, which showed lower values until d8, compared to d18 (*p* < 0.001). However, in T piglets, d8 and d18 were not significantly different anymore (d3–d5 vs. d18 *p* < 0.001). *l*_stride_′ was consistently shorter in P piglets (*p* < 0.001 at all ages). *f*′ showed a fairly inconsistent maturation pattern in T piglets with d3 and d8 (but not d4 and d5) being significantly different from the reference age at d18 (*p* < 0.001 and *p* = 0.014, respectively). This gait characteristic did not show maturational changes in P piglets. At d3, d5, and d8, *f*′ was significantly higher in P piglets, compared to T piglets (*p* < 0.001, *p* = 0.018, 0.004, respectively) (Figure 2B) (data points = 860).

While *u*′ did not differ between P-LBW (*n* = 9) and P-NORM (*n* = 9) (Figure 2C) (data points = 60), *f*′ was overall higher for P-LBW (*p* = 0.003), while *l*_stride_′ was higher for P-NORM (*p* < 0.001) (Figure 2D) (data points = 240). *u*′ did not show any age-related changes in P-LBW and P-NORM. However, *l*_stride_′ increased (all comparisons *p* < 0.001) whereas *f*′ discretely dropped (d3 vs. d18: *p* = 0.003) (Figure 2D).

Similarly, *u*′ did not differ between P-MALE (*n* = 21) and P-FEMALE (*n* = 17) piglets and did not differ with postnatal age (Figure 2E) (data points = 134). *f*′ was higher (*p* = 0.001) whereas *l*_stride_′ (at d3, 8, and 18; *p* < 0.001, 0.018, 0.037, respectively) was lower for P-MALE, compared to P-FEMALE. *l*_stride_′ increased with age in both P-MALE and P-FEMALE (all comparisons with d18, *p* < 0.001) (Figure 2F) (data points = 536).

### Neuromotor Control—Normalized Gait Characteristics

For *df*, we noted a short window of maturation, evidenced by the larger value at d3, compared to d18 (*p* < 0.001) (Table 4). *Df* was lower for P-MALE (*n* = 21), compared to P-FEMALE (*n* = 17) (*p* = 0.019).

**Table 4:** Duty factor (*Df*) (mean ± SD; m) according to gestational age when cesarean-section derived (condition) (preterm at gestational age 106 days—term at gestation age 115 days) and postnatal age (3, 4, 5, 8, and 18 days), birth weight (low birth weight (LBW): 0–25th percentile—normal birth weight (NORM): 50–75th percentile), and sex (female vs. male).

	**Effect**					
	**Condition**		**Birth weight (preterms)**		**Sex (preterms)**	
**Age**	**Preterm**	**Near term**	**LBW**	**NORM**	**Female**	**Male**
d3	0.64 ± 0.08^a^	0.68 ± 0.09^a^	0.60 ± 0.08	0.61 ± 0.09	0.65 ± 0.08	0.63 ± 0.09
d4	0.63 ± 0.12^a^^,^^b^	0.65 ± 0.10^a^^,^^b^	0.64 ± 0.07	0.64 ± 0.12	0.65 ± 0.11	0.62 ± 0.12
d5	0.63 ± 0.09^a^^,^^b^	0.64 ± 0.07^a^^,^^b^	0.62 ± 0.09	0.66 ± 0.09	0.64 ± 0.08	0.63 ± 0.09
d8	0.63 ± 0.11^a^^,^^b^	0.61 ± 0.01^a^^,^^b^	0.61 ± 0.11	0.62 ± 0.12	0.66 ± 0.12	0.61 ± 0.11
d18	0.64 ± 0.07^b^	0.63 ± 0.05^b^	0.64 ± 0.04	0.66 ± 0.07	0.65 ± 0.07	0.63 ± 0.06

*There was no interaction between condition, sex, or birth weight and postnatal age. Df was higher in P-FEMALE when compared with P-MALE*.

a−b *Different superscripts indicate significant differences with postnatal age within condition, birth weight, or sex*.

**Figure 3:**
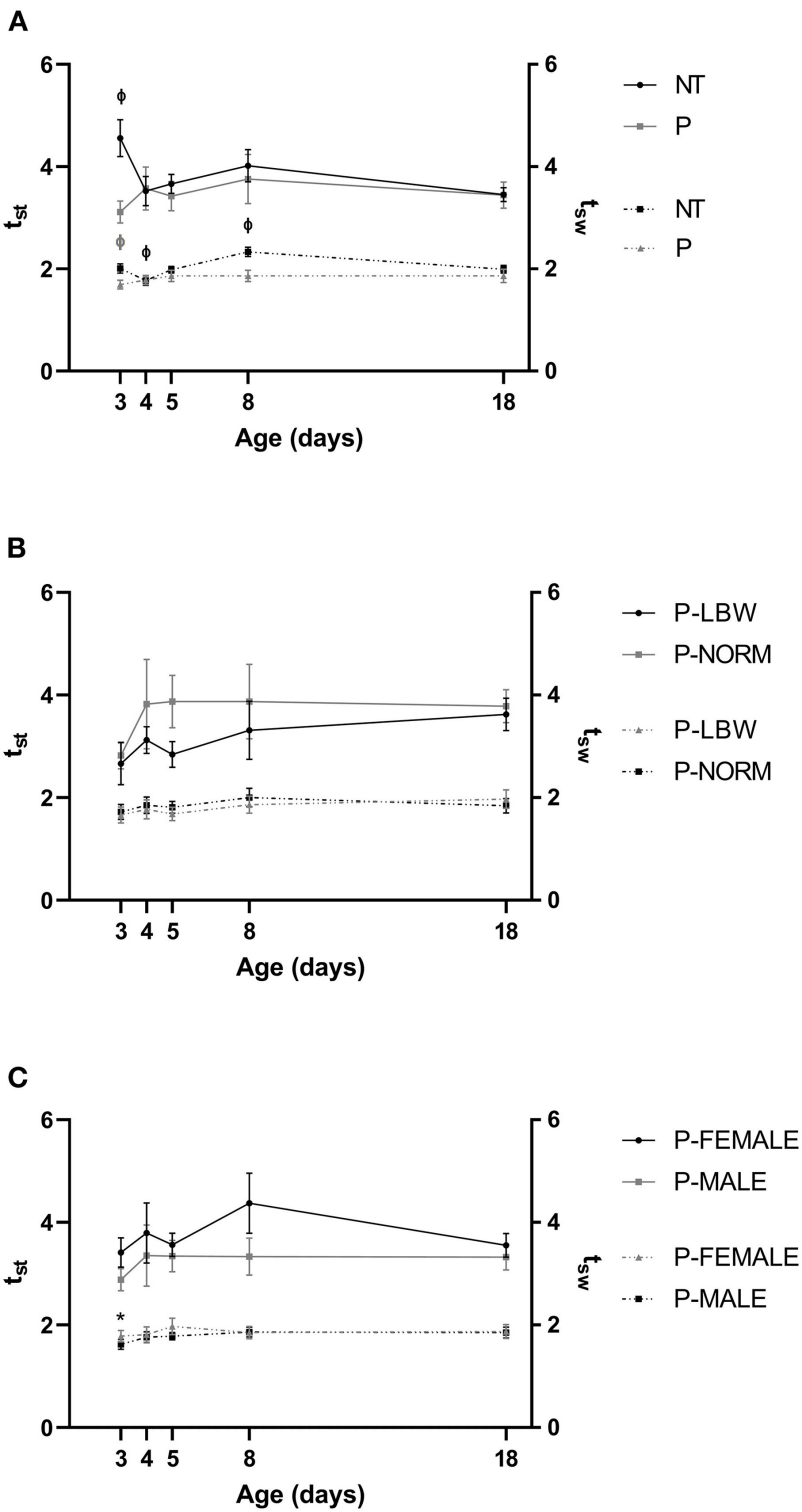
Normalized stance and swing duration. All values are mean ± SE. **(A)** Normalized stance duration ($t_{\mathrm{st}}^{\prime }$; full line) and swing duration ($t_{\text{sw}}^{\prime }$; dotted line) according to condition (P: preterm, gray; T: term, black) and age. Mean values for $t_{\mathrm{st}}^{\prime }$ at d3 and *t*_sw_′ at d3, d5, d8, and d18 were higher in T than in P. Mean values of $t_{\mathrm{st}}^{\prime }$ and *t*_sw_′ indicated with “Φ” were different from d18. **(B)** Normalized stance duration ($t_{\mathrm{st}}^{\prime }$; full line) and swing duration ($t_{\text{sw}}^{\prime }$; dotted line) according to birth weight (normal birth weight (P-NORM); low birth weight (P-LBW) and postnatal age. $t_{\mathrm{st}}^{\prime }$ was higher in P-NORM compared to P-SGA. **(C)** Normalized stance duration ($t_{\mathrm{st}}^{\prime }$; full line) and swing duration ($t_{\text{sw}}^{\prime }$; dotted line) according to sex (female: P-FEMALE; male: P-MALE) and postnatal age. $t_{\mathrm{st}}^{\prime }$ was higher in P-FEMALE compared to P-MALE. Mean values of *t*_sw_′ indicated with “*” were different from d18. Black colors indicate differences in T while gray colors indicate differences in P piglets.

The mean value of $t_{\mathrm{st}}^{\prime }$ was similar in both groups except at d3, when it was higher for T piglets (*n* = 20) compared to P piglets (*n* = 38) (*p* < 0.001). Mean *t*_sw_′ was lower for P than for T piglets at most time points (*p* < 0.001 at d3 and d8, *p* = 0.017, 0.033 at d5 and d18) (Figure 3A) (data points = 860). In T piglets, $t_{\mathrm{st}}^{\prime }$ was significantly higher at d3 compared to d18 (*p* = 0.002), while there was no maturation visible in P piglets. *t*_sw_′ had a longer window of maturation in T piglets, with both d4 and d8 exhibiting significantly higher values compared to d18 (*p* < 0.001, for both). In P piglets, *t*_sw_′ for d3 was lower when compared with d18 (*p* = 0.010). $t_{\mathrm{st}}^{\prime }$ was higher in P-NORM (*n* = 9) compared to P-LBW (*n* = 9) (*p* = 0.034) (Figure 3B) (data points = 240) and for P-FEMALE (*n* = 17) compared to P-MALE (*n* = 21) (*p* = 0.001) (Figure 3C) (data points = 536) while *t*_sw_′ did not differ. As observed in the comparison between T and P piglets, $t_{\mathrm{st}}^{\prime }$ did not show any differences with postnatal age, while the effect of postnatal age on $t_{\text{sw}}^{\prime }$ was confirmed when comparing P-FEMALE with P-MALE (*p* = 0.004).

*l*_step_′ was higher in T pigs (*n* = 20) compared with P (*n* = 38) pigs (*p* < 0.001) while *h*_swmax_′ was similar. *l*_step_′ increased (all comparisons: *p* < 0.001) whereas *h*_swmax_′ dropped with postnatal age (all comparisons: *p* < 0.001). The latter occurred in a leg-dependent manner: *h*_swmax_′ was higher at d3, d4, d5, and d8, compared to d18 for LF, RF, and LF. For RF, d8 did not differ from d18 anymore. *l*_step_′ was overall higher for P-NORM (*n* = 9) (*p* < 0.001) while $h_{\text{swmax}}^{\prime }$ did not differ between P-LBW (*n* = 9) and P-NORM. *l*_step_′ (d3, d8, d18; *p* < 0.001, *p* < 0.001, *p* = 0.001, respectively) and $h_{\text{swmax}}^{\prime }$ (*p* = 0.001) were lower for P-MALE (*n* = 21), compared to P-FEMALE (*n* = 17) (Table 5).

**Table 5:** ormalized step length (*l*_step_′) and maximal swing height (*h*_swmax_′) (mean ± SD; m) according to gestational age when cesarean-section derived (preterm at gestational age 106 days—term at gestation age 115 days) and postnatal age (3, 4, 5, 8, and 18 days), birth weight (low birth weight (LBW): 0–25th percentile—normal birth weight (NORM): 50–75th percentile), and sex (female vs. male).

	**Effect**					
	**Condition**		**Birth weight (preterms)**		**Sex (preterms)**	
**Age**	**Preterm**	**Near term**	**LBW**	**NORM**	**Female**	**Male**
***l*_*step*_′**						
d3	0.53 ± 0.14^a^	0.65 ± 0.12^a^	0.36 ± 0.09^a^	0.55 ± 0.14^a^	0.59 ± 0.11^a^	0.48 ± 0.14^a^
d4	0.55 ± 0.12^a^	0.69 ± 0.15^a^	0.51 ± 0.12^a^	0.58 ± 0.14^a^	0.57 ± 0.13^a^	0.53 ± 0.11^a^
d5	0.58 ± 0.13^a^	0.71 ± 0.12^a^	0.49 ± 0.13^a^	0.62 ± 0.13^a^	0.59 ± 0.11^a^	0.58 ± 0.14^a^
d8	0.62 ± 0.15^a^	0.78 ± 0.15^a^	0.53 ± 0.13^a^	0.67 ± 0.18^a^	0.68 ± 0.12^a^	0.59 ± 0.15^a^
d18	0.75 ± 0.09^b^	0.84 ± 0.12^b^	0.74 ± 0.10^b^	0.76 ± 0.08^b^	0.78 ± 0.11^b^	0.72 ± 0.07^b^
***h*_*swmax*_′**						
d3	0.14 ± 0.05^a^	0.14 ± 0.04^a^	0.15 ± 0.06^a^	0.14 ± 0.05^a^	0.15 ± 0.05^a^	0.13 ± 0.05^a^
d4	0.14 ± 0.05^a^	0.13 ± 0.04^a^	0.13 ± 0.05^a^	0.13 ± 0.04^a^	0.14 ± 0.05^a^	0.14 ± 0.05^a^
d5	0.14 ± 0.05^a^	0.14 ± 0.04^a^	0.13 ± 0.05^a^	0.14 ± 0.06^a^	0.16 ± 0.06^a^	0.13 ± 0.05^a^
d8	0.12 ± 0.05^a^	0.14 ± 0.04^a^	0.13 ± 0.05^a^	0.13 ± 0.05^a^	0.13 ± 0.06^a^	0.12 ± 0.04^a^
d18	0.09 ± 0.04^b^	0.09 ± 0.03^b^	0.08 ± 0.03^b^	0.08 ± 0.03^b^	0.10 ± 0.05^b^	0.09 ± 0.04^b^

*$l_{\text{step}}^{\prime }$ was higher in T pigs compared with P pigs, in P-NORM compared to P-SGA and in P-FEMALE compared with P-MALE. $l_{\text{step}}^{\prime }$ differed with postnatal age. $h_{\text{swmax}}^{\prime }$ was higher in P-FEMALE when compared with P-MALE and dropped with postnatal age*.

a−b *Different superscripts within a column indicate significant differences with postnatal age within condition, birth weight, or sex*.

### Gait Symmetry

**Figure 4:**
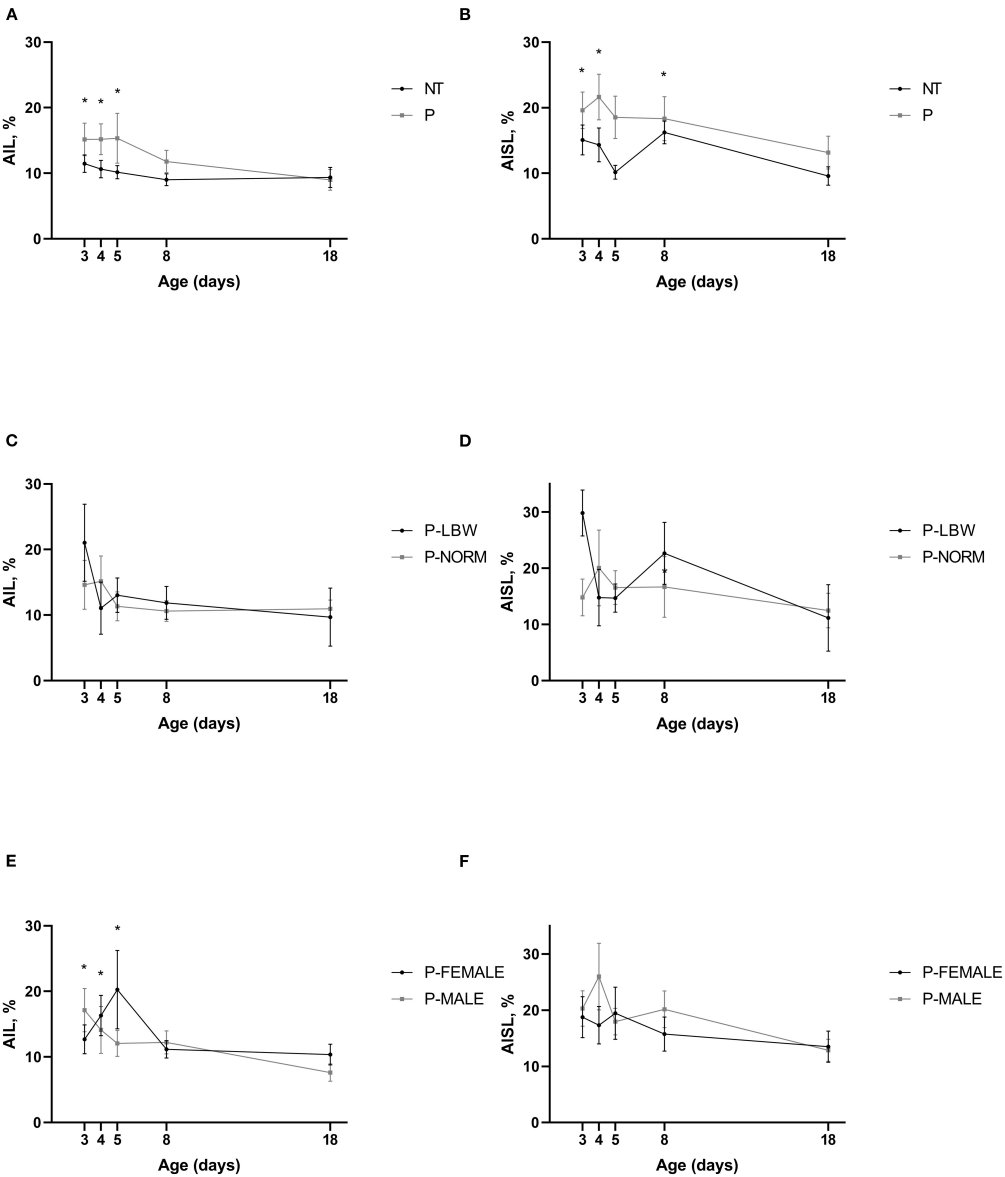
Gait symmetry. All values are mean ± SE. Asymmetry index stride length (AIL) according to postnatal age and **(A)** condition (P: preterm, gray; T: term, black), **(B)** birth weight (normal birth weight (P-NORM); low birth weight (P-LBW), and **(C)** sex (female: P-FEMALE; male: P-MALE). AIL is higher in P than in T piglets. Mean values indicated with * differ significantly from d18. Asymmetry index step length (AISL) according to postnatal age and **(D)** condition (P: preterm, gray; T: term, black), **(E)** birth weight (normal birth weight (P-NORM); low birth weight (P-LBW), and **(F)** sex (female: P-FEMALE; male: P-MALE). AISL is higher in P than in T piglets (front leg). Mean values indicated with * differ significantly from d18.

P piglets (*n* = 38) showed a higher AIL (*p* < 0.001) and AISL (only the front legs, *p* < 0.001), compared to T piglets (*n* = 20). AIF, AIST, AISW, and AIDF did not differ between P and T piglets. There was some maturation visible for several of the asymmetry variables (similar in T and P piglets). AIL was significantly higher at d3, d4, and d5 compared to d18 (*p* = 0.008, 0.019, 0.028, respectively) (Figure 4A) (data points = 430). A similar observation was seen when looking at P-MALE (*n* = 21) and P-FEMALE (*n* = 17) (*p* = 0.031) (Figure 4) (data points = 268) but not in the preterm pigs belonging to selected birth weight categories (Figure 4) (*n* = 18, data points = 120). Higher values for AISL were noted at d3, d4, and d8, compared to d18 (*p* = 0.008, 0.005, 0.011, respectively) (Figure 4D). For AISW and AIDF, only d8 was significantly higher than d18 (*p* = 0.002, 0.002, respectively). Only one of the asymmetry indices differed between P-LBW (*n* = 9) and P-NORM (*n* = 9) piglets. AIF was lower for P-LBW at d4 and d18 (*p* = 0.028, 0.041, respectively). None of the asymmetry indices were different between P-MALE (n = 21) and P-FEMALE (*n* = 17) piglets (Figure 4).

## Discussion

### The Effect of Premature Birth

As expected, both body weight and HLL were lower in P compared to T piglets during the first week of postnatal life. This indicates that when the preterms are born, they are indeed smaller due to a shortened period of growing *in utero*. However, by d18 this difference disappeared, showing that P piglets catch up with T piglets after a week.

When looking at motor performance (measured by *u*), P and T piglets performed equally well. This was somewhat surprising since, given their lower muscle mass and lesser muscle anabolic response (31), we expected that preterm pigs would be overall slower. However, it is possible, that, relative to total body weight, their muscle mass is equally (or more) developed. We have found this to be true for low birth weight piglets in our previous studies (24, 32), and this is also the case in preterm piglets vs. their term counterparts at 26 days of age (14). Thus, their muscle mass might allow them to produce enough force to keep up their motor performance (voluntary self-selected speed), which would translate into a higher frequency that counterbalances the smaller strides of P piglets. Additionally, the lack of a difference in motor performance might indicate that gait performance has little to do with post-conceptional age in late gestation, but more with postnatal age and environmental factors. Not only did both groups remain in a heated incubator for 5 days before being transferred to an open cage, but also they received the same combination of enteral/parenteral food (relative to body weight), taking a possible difference in feed intake or route of administration (enteral vs. parenteral) that may affect gait development (10, 32) out of the equation.

Overall neuromotor maturation (*u*′) was not delayed in P piglets and reached maturity between d5 and d8 in both P and T piglets. This supports our hypothesis that postnatal age and environment, rather than post-conceptional age, affect neuromuscular development. At first sight, our results are surprising, because other studies have demonstrated that preterm pigs show motor coordination delays during the first weeks after birth (10, 14, 33). According to Andersen et al. (14), these delays are generally shorter than the reduction in gestation length, which indicates that preterm pigs do show some developmental plasticity. In their study, piglets were born 12 days preterm, but basic motor function (first time standing up and first time walking), locomotion, and balance/coordination scores suggested delays of 2, 5, and 11 days, respectively. A delay of 2–3 days in basic motor function in preterm vs. term piglets was confirmed by Obelitz-Ryom et al. (11). In this study, the majority of both preterm and term piglets were standing up and walking by the third postnatal day. A delay in the onset of walking (1.43–2.16 months) is seen in preterm infants (6) and rabbits (34). In rabbits, the lower motor score observed in preterm (28 days of post-conceptional age) vs. term (31 days post-conceptional age) newborns corresponded with lower neuron densities in the former (34). As such, it is likely that we “missed” these delays in our motor performance and neuromotor development data, because recordings were only made from day 3 onward and precocial animals show a relative mature brain and neuromuscular functions by the end of gestation (10, 35).

When looking into the neuromotor skills (normalized gait characteristics), P and T piglets have different strategies to reach a mature neuromotor performance if we consider normalized speed as the proxy for the latter. This differing repertoire is in line with several studies in preterm infants, for example, (1, 6). Different patterns of neuromotor skills' development were observed in our study: 1) no maturation in P piglets (*f*′, $t_{\mathrm{st}}^{\prime }$), 2) slightly longer maturation ($l_{\text{stride}}^{\prime }$$t_{\mathrm{st}}^{\prime }$ ), or 3) similar pattern of maturation (*df*, $l_{\text{stride}}^{\prime }$) when compared to T piglets. Based on these results, one might consider preterm pigs to suffer from a “stunted” maturation for certain aspects of the gait that is compensated for by other neuromotor skills. This is reflected in a differently looking gait at the age of 18 days in P piglets irrespective of their body dimensions: P piglets take shorter steps (indicated by shorter $l_{\text{stride}}^{\prime }$, shorter $l_{\text{stride}}^{\prime }$, shorter time between footfalls shown by shorter $t_{\text{sw}}^{\prime }$) at a higher frequency (indicated by an overall higher *f*′) than T piglets. In preterm infants during childhood, a shorter stride length was also reported (6). As neuromotor maturation is completed by d18 (23, 24), it is likely that this difference in neuromotor skills (gait characteristics) between P and T piglets remains during the rest of their life. A follow-up study in adult pigs is needed to confirm this as in a more detailed study focusing on 3–5 postnatal age. Such a study—including challenges such as hurdles and treadmills—can provide more insight in the “gait repertoire” of preterm vs. term pigs of which this study hints that this is differing.

Taking a closer look at gait variability, we compare the balance/coordination results of Andersen et al. (14) with our results, which are strikingly different. Where they suggest a delay of 11 days for preterm pigs, compared to term pigs, we see no difference between groups in achieving a symmetrical gait pattern. The most likely explanation is the different way of studying balance/coordination between Andersen et al. (14) and our study. Where we digitized and calculated AIs, they relied on scoring by the experimenters. We observed that both T and P pigs took a fairly long time to achieve a symmetrical gait, with some variables only reaching a stable value between d8 and 18. This was expected for P piglets. However, we expected T piglets to have an already mature state at d3, as we know from a previous study that term piglets achieve gait symmetry within the first day after birth (23, 24). Why this is not the case in this study might be explained by the different rearing conditions and “exercise” in these studies, where in Vanden Hole et al. (23, 24) the piglets were vaginally delivered, did not receive artificial feeding, and remained with the sow in a farm environment. The postnatal environment also proved its importance in rat pups, which suffer from abnormal locomotion that lasted even until adulthood when rats were subjected to both prenatal (i.e., intrauterine hypoperfusion) and postnatal (i.e., sensorimotor restriction) insults (36). Studies on gait symmetry (and stability) in human preterms are inconclusive on whether or not a difference exists and whether this is maintained throughout development (6).

### The Effect of LBW

The body weight of both groups of preterm piglets increased over time, but the difference in body weight at birth between P-LBW and P-NORM piglets was maintained, indicating that P-LBW piglets do not show catch-up growth, relative to their normal-size preterm littermates, within the first 18 days. However, this difference in body weight resulting from fetal growth restriction did not affect motor performance, indicating that, relative to total body weight, muscle mass in P-LBW and P-NORM piglets is similar. It could be expected that in P-LBW piglets the energy reserve at birth is lower, as we also found in term LBW pigs (32). In term LBW pigs, this is probably the main reason for the lower performance in growth-restricted animals (24). However, in our current study, the lesser energy reserves are replenished rapidly by the artificial rearing and pigs are assessed at a later age.

P-LBW and P-NORM piglets also show the same pattern of neuromotor maturation in all of the investigated variables. With regard to *f*′, *l*_stride_′, and *l*_step_′, a difference in neuromotor skill was detected. However, it must be noted that by d18 differences are reduced to a minimum, implying that the gait looks the same in P-LBW and P-NORM. The lack of visible differences in neuromotor maturation is possibly due to the lack of video recordings during the first 3 days, a period where other studies have reported delays in basic neuromotor skill (11, 14). Most of the pigs in these studies were capable of standing up and walking within the first 3 days after birth. In addition, the delays in first-time standing up and first-time walking were only 15 and 17 h, respectively. In order to register these fairly short delays, a future study should keep in mind that time points for gait measurements should be closer together and start at the time of first-time walking. In a previous study, detailing neuromotor development in the first 4 days in low birth weight vs. normal birth weight term piglets, we found no differences in time of maturation, but we did find a difference in neuromotor skill (more specifically, *f*′, *t*_sw_′, and $t_{\mathrm{st}}^{\prime }$) (24). With the exception of AIF, gait variability was the same in P-LBW and P-NORM piglets. These results are in line with previous results on gait variability in low birth weight and normal birth weight term piglets, where also no difference in gait variability was detected (24).

Overall, our results suggest that growth restriction hardly affects gait development in preterm pigs between 3 and 18 days of life when artificially reared.

### The Effect of Sex

Body weight and HLL were not different in P-MALE and P-FEMALE piglets across the entire studied period. In agreement with their body dimensions (similar leg length, muscle mass) and the same artificial rearing scheme, motor performance was the same in both groups. Neuromotor maturation shows the same pattern in P-MALE and P-FEMALE piglets, but neuromotor skill often differed between sexes (with the exception of u′). Most variables showed a larger mean value in P-FEMALE, except for f′ which was higher in P-MALES. It is important to keep in mind though that these differences in neuromotor skill do not imply a better or worse gait pattern in either sex. This just means that their gait, relative to their body dimensions, is slightly different, an observation we also saw when comparing P with T piglets. Simply put, whereas P-MALES take more, but shorter, steps to cover a certain distance, P-FEMALES will take fewer, but larger steps. Gait variability was the same in both groups across the entire studied period. These fairly limited differences in gait between sexes are consistent with Bæk et al. (20) and with our previous studies on term piglets (23) where no sex-related differences regarding overall motor performance were reported.

### Relevance

Piglets born at 90% gestation are often considered to have an overall survival capacity and gut function of 28–30-week-old infants [see review by (8)], while the developing brain may be more similar to 34–37 week old infants, the so-called “late preterm infants” (14, 33). In agreement with this, the development of the skeletal and nervous systems is considered to be slightly faster in newborn piglets than in babies, as reviewed elsewhere (37, 38). As such, our results will be most relevant for the latter category of preterm infants. These late preterm infants have only recently become a topic of interest. Although they comprise the bulk part of preterm births [considering the increasing number of elective cesarean sections (39), they remained fairly unrepresented in studies because, compared to early preterm infants, their deficiencies (after birth and later in life) seemed less severe (1, 2, 4, 6, 40). However, it has become clear that late preterm infants experience a substantial mortality and neonatal morbidity [see review by (41)]. Compared to term infants, they exhibit higher rates of temperature instability, respiratory distress, hypoglycemia, jaundice, etc. (42–44). Also, later in life, these infants experience difficulties in speaking, writing, mathematics, behavior, and physical education and are at higher risk for motor impairment (1, 2, 4, 6, 45). In our study, we put the preterm piglet forward to study the effect of late prematurity on neuromotor skill development. Our results show that the precocious nature of the pig with regard to neuromotor development is preserved in case of preterm birth since most of the effects of prematurity resolved by days 3–5. Thus, when studying effects of gestational age, birth weight, sex, and postpartum environment (nutritional interventions, exercise) on neuromotor development using the preterm piglet, the focus should lay on the first days after delivery. It must be considered that pigs delivered prematurely were not able to stand and walk within the first hours after life. It was only on day 3 when most of the piglets included in this study were able to walk and therefore be recorded. This may be a limitation of this model, since the first postnatal hours are critical in the development of neuromotor skills. In addition, the results show that neuromuscular development—as seen for other organs systems—is highly plastic and capable to catch up, showing that postnatal rather than post-conceptional age is the main driver. This indicates that there is a window of opportunity to optimize the neuromotor performance in the case of prematurity. In addition, the gait of the preterm pig shares many characteristics (shorter stride length, delayed-onset first walking, reduced repertoire) with that of the human infant, putting the preterm pig at the forefront as a translational model.

## Data Availability Statement

The original contributions presented in the study are included in the article/supplementary material, further inquiries can be directed to the corresponding author/s.

## Ethics Statement

The animal study was reviewed and approved by National Ethics Committee on Animal Experimentation (protocol no. 2012-15-2934-00193).

## Author Contributions

TT and PS conceived and designed the piglet experiment. PA, CVand, and CVan conceived and designed the gait analysis experiment. CVand collected the data and performed the analysis. CVan, SV, PA, TT, and PS contributed the data and/or analysis tools. All authors discussed the results and contributed to the final manuscript. CVand, CVan, and MA provided the graphs and translated these discussions into the paper.

## Funding

This work was supported by the University of Antwerp (grant number GOA-33927). The experimental work was funded by Arla Foods Ingredients and the Innovation Fund Denmark (NEOMUNE) grant. The authors declare that this study received funding from Arla Foods Ingredients. The funder was not involved in the study design, collection, analysis, interpretation of data, the writing of this article or the decision to submit it for publication.

## Conflict of Interest

The authors declare that the research was conducted in the absence of any commercial or financial relationships that could be construed as a potential conflict of interest.

## Publisher's Note

All claims expressed in this article are solely those of the authors and do not necessarily represent those of their affiliated organizations, or those of the publisher, the editors and the reviewers. Any product that may be evaluated in this article, or claim that may be made by its manufacturer, is not guaranteed or endorsed by the publisher.

## Abbreviations

AIDF: AI duty factor
AIF: AI stride frequency
AIL: AI stride length
AISL: AI step length
AIST: AI stance duration
AISW: AI swing duration
*df*: duty factor
*f*: stride frequency
*g*: gravitational acceleration
*h*_*swmax*_: maximum swing height
HLL: hind limb length
*l*_step_: step length
*l*_stride_: stride length
*t*_*st*_: stance duration
*t*_*sw*_: swing duration
*u*: self-selected speed.
